# Antibody-Integrated Solid-to-Gel Microfilm for Protection Against Botulinum Neurotoxin Type A

**DOI:** 10.3390/gels11100777

**Published:** 2025-09-27

**Authors:** Ji-Hwan Ha, Sohee Jeon, Yun-Woo Lee, Soon Hyoung Hwang, Byung-Ho Kang, Young Jo Song, Ji-Su Lim, Hyunbeen Kim, Yoosik Yoon, Jun-Ho Jeong

**Affiliations:** 1 Nano Lithography and Manufacturing Research Center, Korea Institute of Machinery and Materials (KIMM), Daejeon 34103, Republic of Korea; jhha@hanbat.ac.kr (J.-H.H.); sjeon@kimm.re.kr (S.J.); yunwoo417@ssu.ac.kr (Y.-W.L.); soon814@kimm.re.kr (S.H.H.); royce2080@kimm.re.kr (B.-H.K.); 2 Department of Mechanical Engineering, Hanbat National University, Daejeon 34158, Republic of Korea; 3 College of Pharmacy, Chung-Ang University, Seoul 06974, Republic of Korea; 4 Department of Mechanical Engineering, Soongsil University, 369 Sangdo-ro, Dongjak-Gu, Seoul 06978, Republic of Korea; 5 The 3rd R&D Institute, Agency for Defense Development, Daejeon 34186, Republic of Korea; 6 College of Medicine, Chung-Ang University, Seoul 06974, Republic of Korea; earth9910@naver.com (J.-S.L.); hb1hb@naver.com (H.K.)

**Keywords:** BoNT/A protection, antibody-integrated solid-to-gel microfilm, anti-BoNT/A antibody, gel-integrated antibody, antibody microfilm-coated needles

## Abstract

Antibodies are indispensable for protection against biological toxins and pathogens, yet their conventional liquid formulations impose severe constraints, including dosing inaccuracy caused by residual fluid remaining in the syringe and limited user convenience such as pain caused by fluid-induced tissue distension and nerve stimulation as well as instability in ambient temperature, and the requirement for low-temperature storage and logistics. These limitations critically impair rapid deployment during golden hour following acute exposure. Here, we report an antibody-integrated solid-to-gel microfilm—demonstrated with a 100 µg anti-BoNT/A dose—jet-printed and low-temperature dried directly onto metal needles for consistent, on-demand use. Upon intradermal insertion, the microfilm fully dissolves within 5 min, driven by hydration-induced swelling of a hyaluronic acid (HA) support layer and rapid release of the antibody. Time-resolved microscopy and UV–vis analysis showed a decrease in residual solid from 2.34 mm^3^ to 0 over 300 s, with a concomitant rise at 187 nm indicative of complete dissolution. The solid formulation maintained ambient-temperature stability for 3–6 months with pharmacokinetics comparable to conventional subcutaneous liquid injections. In a lethal BoNT/A challenge, treated mice achieved 100% survival for 12 days, whereas controls succumbed within 16 h.

## 1. Introduction

The development of antibodies capable of neutralizing diverse biological toxins and protecting against pathogens has been an enduring focus in biomedical research, driven by the need to safeguard public health in modern society [1,2,3]. Antibodies function by binding to specific antigens, thereby neutralizing their activity or eliciting protective immunity [4]. Traditionally, antibodies are stored and administered in liquid form to facilitate immediate use [2]. However, liquid formulations present inherent drawbacks, including dosing inaccuracy caused by residual fluid remaining in the syringe and limited user convenience such as pain caused by fluid-induced tissue distension and nerve stimulation as well as instability in ambient temperature, and the requirement for low-temperature storage and logistics [5]. These limitations severely compromise their utility in time-critical scenarios—such as acute toxin exposure, battlefield injuries, or civilian medical emergencies—where rapid, accurate, and reliable administration is essential. This underscores the need for antibody delivery platforms that are stable, user-friendly, logistically efficient, and deployable on demand.

Botulinum neurotoxin (BoNT) is among the most potent toxins known [6,7,8], produced by *Clostridium botulinum*, an anaerobic, Gram-positive, spore-forming bacterium. Eight serotypes (A–H) have been identified, with BoNT type A (BoNT/A) being a primary cause of severe botulism. This toxin can be acquired through contaminated food, wound infection, or inhalation; remarkably, inhalation of only 0.7–0.9 μg or ingestion of 70 μg can be lethal to a healthy adult. BoNT inhibits acetylcholine release at neuromuscular junctions, causing acute flaccid paralysis that progresses to respiratory failure through paralysis of the pharynx, diaphragm, and intercostal muscles [9,10]. Current treatment for BoNT intoxication primarily relies on antiserum. In our previous research, we developed humanized monoclonal antibodies (mAbs) for botulism therapy, which differ fundamentally from antiserum in both antibody composition and efficacy, as the latter depends on the immunization of horses or humans [11,12]. Without prompt intervention, death is inevitable. mAbs targeting BoNT/A have demonstrated protective efficacy in mice and rabbits, offering advantages over equine-derived antitoxins due to reduced adverse effects [13]. Nevertheless, existing anti-BoNT/A antibody therapies remain dependent on liquid formulations and conventional injection methods, inheriting the limitations of traditional antibody delivery. Alternative antibody delivery systems such as dissolving microneedles, nanoparticles, and implantable gels have been explored [14] but limitations including long dissolution times, cold-chain dependence, and invasiveness remain.

In this work, we present a solid-to-gel microfilm-coated needle-based delivery system for anti-BoNT/A antibodies with hyaluronic acid (HA) gel designed to overcome these constraints of conventional liquid-state antibodies. The approach involves quantitatively loading, anchoring, and low-temperature drying antibody solutions directly onto metal needles with micro diameters, enabling consistent dosing and robust adhesion. HA is well known to undergo rapid hydration-induced swelling, forming a viscoelastic gel-like matrix upon contact with aqueous environments [14,15]. The dried HA layer applied onto metal needles was designed to exploit this property, enabling a solid-to-gel transition in situ after skin insertion. This swelling-driven gelation provides a biocompatible support that not only anchors the antibody microfilm but also facilitates its rapid dissolution and diffusion. Upon skin injection, the solidified antibodies dissolve completely within 5 min, ensuring rapid therapeutic action. A precision jetting system for scalable production, enabling the reproducible application of uniform antibody quantities and customization to specific operational requirements, was developed. Structural analyses were performed to investigate morphological changes during drying and skin injection, while dissolution kinetics and long-term stability were evaluated up to six months. In vivo studies using a mouse model confirmed that the BoNT/A antibody-integrated solid-to-gel microfilm retains their bioactivity, validating this platform as a promising strategy for rapid, stable, and user-friendly countermeasures against BoNT and other high-risk toxins.

## 2. Results and Discussion

### 2.1. Research Concept

**Figure 1:**
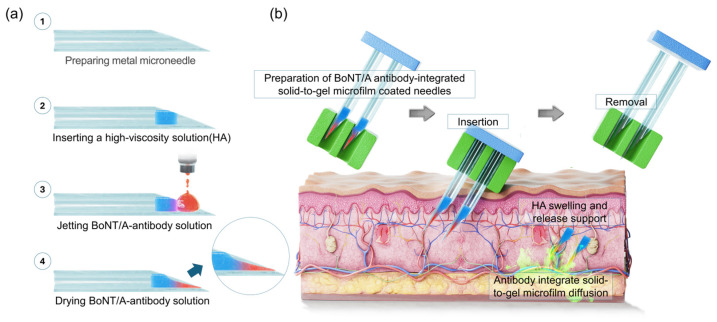
Illustration of solid-state antibody fabrication and application overview. (**a**) Fabrication of the BoNT/A antibody-integrated solid-to-gel microfilm-coated metal needles (23G; inner diameter 340 μm, outer diameter 640 μm). (**b**) Overview of strategies for applying the antibody-integrated solid-to-gel microfilm-coated needles. (HA: hyaluronic acid).

The solidification of antibodies requires the precise and reproducible deposition of liquid droplets onto metal needle surfaces. To achieve this, we developed a high-precision jetting system capable of continuously dispensing a constant antibody volume onto metal needles. Figure 1a outlines the fabrication process. (i) Metal needles were first prepared as the delivery platform. (ii) A biocompatible hyaluronic acid (HA) solution [16,17,18], known to promote skin cell regeneration [19,20], was applied to the needles. Driven by capillary action, the HA solution infiltrated the needle channels and subsequently gelified upon drying. (iii) Antibody solution droplets of uniform volume were then dispensed onto the tip region of the metal needles using the jetting equipment. (iv) Following low-temperature drying in a refrigerated chamber (4 °C), repeated cycles of droplet deposition and drying yielded needle surfaces quantitatively loaded with solidified HA and anti-BoNT/A antibodies were conducted. During drying, solvent evaporation induced a characteristic concave morphology within the antibody deposits, a feature that provides structural anchoring advantages when injected into the skin. Upon skin insertion, the pre-dried HA layer rapidly absorbed interstitial fluid and underwent swelling-driven gelation, forming a soft, conformal interface. Such hydration-induced gel-like transitions of HA have been well documented in previous studies on xerogel films and wound-healing hydrogel systems [14,15]. This in situ gelation not only cushioned the insertion site but also facilitated rapid antibody dissolution within 5 min.

Figure 1b illustrates the application process. The antibody-integrated solid-to-gel microfilm is injected into the skin at a shallow angle (~30°) with gentle pressure. Before removal of the metal needles, the HA gel and solid-state anti-BoNT/A antibody rapidly dissolve within the skin and ensure efficient subcutaneous release. The HA matrix concurrently undergoes penetration into skin and dissolution, supporting wound healing and accelerating tissue recovery. Importantly, complete dissolution of the solidified antibodies occurred within five minutes, enabling immediate therapeutic action.

This delivery strategy represents a major departure from conventional liquid antibody administration, offering a dose-accurate and pain-reduced injection, ambient temperature stability without reliance on cold-chain storage and logistics, and user-friendly application. Such advantages render it highly suitable for emergency medicine, battlefield deployment, and civilian exposure of bioterrorism agents. By securing rapid antibody delivery within the critical golden hour, this approach has the potential to significantly improve survival outcomes and broaden the clinical and logistical utility of antibody therapeutics.

### 2.2. Antibody Uniform Jet Process and Solidification

**Figure 2:**
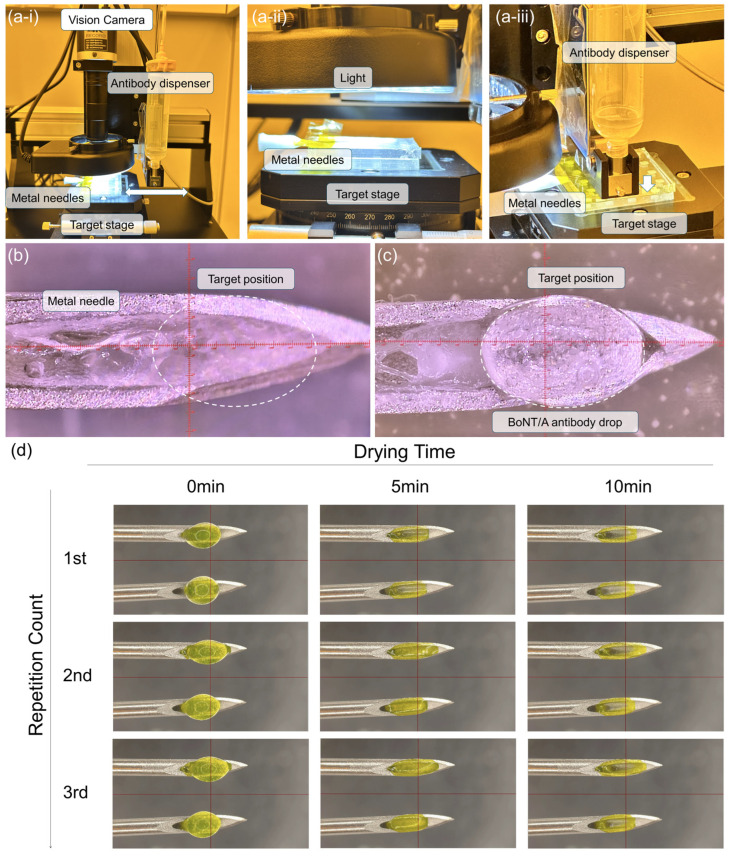
(**a-i**–**a-iii**) Custom-built jetting equipment incorporating a vision system for metal needle positioning and real-time monitoring of droplet applications. (**b**) Metal needle prior to antibody loading, with half of the needles prefilled with HA gel. (**c**) Deposition of anti-BoNT/A antibody solution droplets onto the needle tips using the automated dispenser. (**d**) Time-dependent drying dynamics of the antibody solution at 0, 5, and 10 min, demonstrating solvent evaporation, capillary-driven conformal filling of the needle cavities, and the eventual formation of solidified antibody structures with a concave, coffee-ring morphology after three repeated droplet applications. (HA: hyaluronic acid).

To achieve reproducible HA gelation and antibody solidification, we developed an automated jetting system for the uniform application of anti-BoNT/A antibody solutions. Figure 2(a-i–a-iii) shows the custom-built equipment, which incorporates a vision system for precise metal needle positioning and real-time droplet monitoring. Once the target deposition site is designated, the dispenser moves with micrometer-level accuracy to deliver a predefined volume of anti-BoNT/A antibody solution (100 μg dissolved in 11.96 μL) onto the metal needle surface. The applied droplets are subsequently imaged by the vision system, ensuring accurate alignment and volume control.

Figure 2b displays the metal needle surface prior to the antibody-integrated solid-to-gel microfilm-coated loading, with HA gel pre-filled in half of the needles’ channels to enhance biocompatibility. Following droplet deposition, the anti-BoNT/A antibody solution forms distinct hemispherical droplets at the needle tips (Figure 2c). As the samples are transferred to a refrigerated chamber, the droplets undergo time-dependent solidification driven by solvent evaporation. Figure 2d illustrates this process at 0, 5, and 10 min after application. Initially, droplets maintain a circular geometry, but by 5 min, capillary forces within the needle cavities and surface tension effects guide the drying pattern, conforming to the underlying architecture. At 10 min, complete drying results in solidified antibody structures exhibiting a coffee-ring morphology. Repeated application of three successive droplets onto two identical metal needle surfaces progressively filled indentations and reinforced structural uniformity, producing a concave solid-to-gel microfilm architecture that enhances mechanical anchoring within the skin.

This automated jetting platform ensures uniform antibody deposition, minimizes operator variability, and enables scalable fabrication of antibody-integrated solid-to-gel microfilm-coated needles. Moreover, the characteristic concave morphology generated during drying not only reflects the fundamental interplay between capillary forces and evaporation dynamics but also provides a structural advantage for stable skin retention following injection.

### 2.3. Basic Characteristic and Dissolution Performance of Anti-BoNT/A Antibody-Integrated Solid-to-Gel Microfilm

**Figure 3:**
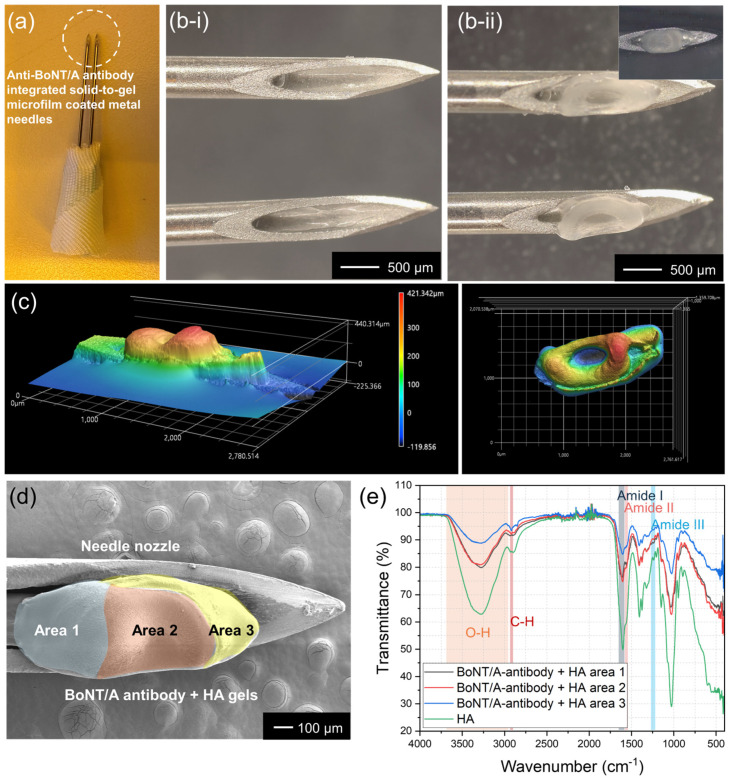
(**a**) Optical image of metal needles uniformly coated with solidified HA gel and anti-BoNT/A antibodies. (**b**) Microscopic images of the needles before (**b-i**) and after (**b-ii**) antibody loading, showing the ring-shaped deposition of solidified anti-BoNT/A antibodies induced by the coffee-ring effect during drying. (**c**) Three-dimensional confocal microscopy of the solidified antibody structure. (**d**) SEM images of HA gel and solidified anti-BoNT/A antibody microfilm on a metal needle, divided into area 1 (blue), 2 (red), and 3 (yellow). (**e**) FTIR analysis of solidified HA and anti-BoNT/A antibodies. Characteristic HA peaks include OH stretching (2900–3600 cm^−1^), CH stretching (~2800 cm^−1^), and Amide I, II, and III bands (1650 cm^−1^, 1550 cm^−1^, 1240 cm^−1^).

Figure 3a presents a real image of metal needles integrated with solidified HA gel and anti-BoNT/A antibodies. The formulation contained anti-BoNT/A antibodies and hyaluronic acid (HA) gel, which sequentially solidify on the metal needle surface (23G; inner diameter 340 μm, outer diameter 640 μm). A magnified microscopic comparison is shown in Figure 3b; prior to antibody loading (i), the needles exhibit a clean surface, whereas after drying and solidification (ii), a distinct ring-shaped deposition of anti-BoNT/A antibodies is observed. This morphology arises from the coffee-ring effect [21,22,23,24] during solvent evaporation, wherein antibody molecules migrate toward the periphery of the droplet, producing a stable annular structure.

The three-dimensional architecture of the solidified antibodies was further characterized using confocal microscopy (Figure 3c). The resulting height profile revealed a maximum peripheral height of 421 μm, while the central indentation measured <100 μm. In top-view mapping, this morphology appeared as a concentric ring, with the lower central region (blue) surrounded by elevated peripheral zones (red). Such concave structures are advantageous for skin anchoring, as they promote mechanical stability and controlled dissolution upon injection.

To investigate spatial distribution, HA gel and anti-BoNT/A antibody coatings were arbitrarily divided into three regions (Figure 3d). FTIR spectroscopy confirmed compositional differences, with area 1 exhibiting characteristic HA peaks, including OH stretching (2900–3600 cm^−1^), CH stretching (~2800 cm^−1^), and Amide I (~1650 cm^−1^), II (~1550 cm^−1^), and III (~1240 cm^−1^) vibrations. From area 1 to 3, HA-associated peak intensities progressively decreased, consistent with an increasing proportion of anti-BoNT/A antibodies localized toward the front portion of the needle. These results collectively confirm that controlled jetting and low-temperature drying produce a structurally reinforced, spatially resolved antibody distribution that supports both stability and functionality within the solid-to-gel microfilm-coated needle system.

**Figure 4:**
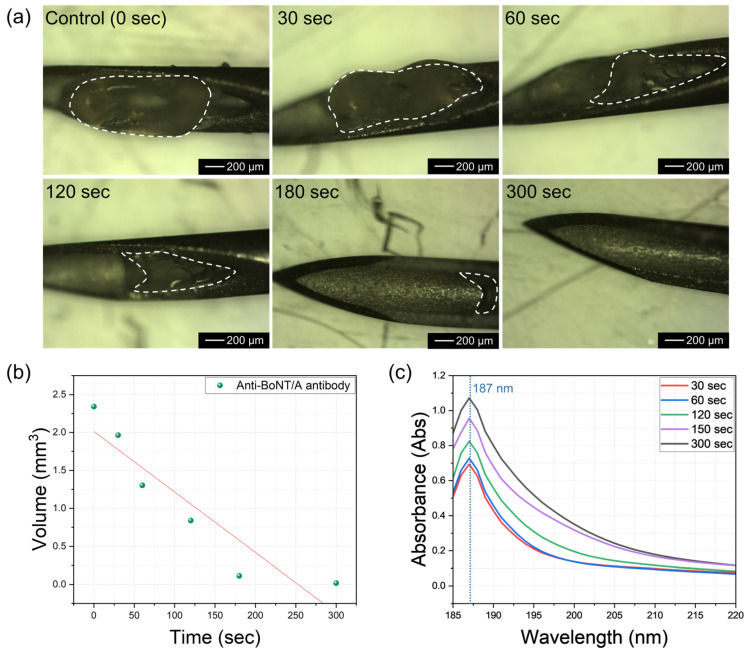
(**a**) Optical microscope images showing the degree of dissolution of solid anti-BoNT/A antibodies as a function of PBS immersion time. (**b**) Change in residual volume of solid anti-BoNT/A antibodies over PBS (37 °C) immersion time. (**c**) UV–vis measurement results of solid anti-BoNT/A antibodies as a function of PBS dissolution time.

Compared with previous dissolving microneedle systems, which typically require 10–20 min for complete dissolution [25,26], the HA-based solid-to-gel microfilm achieved complete release within 5 min, highlighting its superiority for rapid intervention. The dissolution time of this solidified anti-BoNT/A antibody in the body is a very important characteristic for rapid response to BoNT toxins. Considering scenarios where humans need to respond quickly when exposed to bioterrorism agents, this anti-BoNT/A antibody was designed to dissolve completely within 5 min. Figure 4a shows the dissolution characteristics of this solidified anti-BoNT/A antibody over time. When not immersed in PBS solution at 37 °C, the solidified BoNT/A antibody remains intact. However, as the immersion time in PBS solution increases to 30 s, 60 s, 120 s, 180 s, and 300 s, the amount of solidified anti-BoNT/A antibodies remaining on the micro-needles decreases. This suggests that when inserted into the body for 5 min, BoNT/A can be fully delivered through complete dissolution. Figure 4b shows the results of measuring the volume of residual solidified anti-BoNT/A antibodies over time. The initial 2.34 mm^3^ of solid antibody decreased to 1.96 mm^3^ after 30 s, 1.30 mm^3^ after 60 s, 0.83 mm^3^ after 120 s, 0.11 mm^3^ after 180 s, and was completely dissolved after 300 s. Figure 4c shows the UV–vis observation results of the PBS solution in which the solid BoNT/A antibodies were dissolved, as a function of dissolution time. The results showed that as the dissolution time increased, the peak at 187 nm increased, which corresponds to the peak of the protein contained in the antibody. Therefore, PBS containing completely dissolved BoNT/A antibodies at a dissolution time of 5 min exhibited the highest absorbance at 187 nm.

### 2.4. In Vivo Application and Local Skin Response

**Figure 5:**
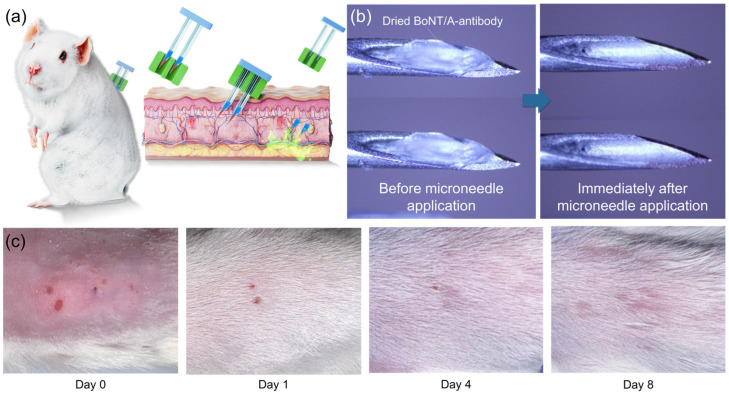
In vivo application and skin response of anti-BoNT/A antibody-integrated solid-to-gel microfilm-coated needles. (**a**) Schematic of the mouse experiment for evaluating anti-BoNT/A antibody-integrated solid-to-gel microfilm-coated needle-mediated antibody delivery. (**b**) Representative images of the microfilm-coated needles before and after skin injection, showing loss of the antibody coating following 5 min of application (blue arrow). (**c**) Photographs of mouse dorsal skin immediately and several days after microfilm-coated needle application.

The in vivo applicability of the BoNT/A antibody-integrated solid-to-gel microfilm-coated needles was confirmed in a mouse model. As illustrated in Figure 5a, the antibody-coated needles were applied to the dorsal skin of mice for 5 min to evaluate skin delivery and tolerability. Previous studies have demonstrated the protective efficacy of humanized anti-BoNT/A antibodies in liquid form [14], whereas our work establishes that the same protective outcome can be achieved with a solid-to-gel formulation that additionally offers logistical and practical advantages. Prior to use, the solid-to-gel microfilm exhibited a uniform antibody coating (Figure 5b, left). After removal, the coating was absent, indicating complete dissolution and release into the skin within 5 min (Figure 5b, right). This observation is consistent with the in vitro dissolution kinetics shown in Figure 4 and demonstrates rapid in vivo release. Local skin examination revealed only minor puncture marks immediately after application (Figure 5c), with no bleeding, erythema, or edema. The marks resolved within 24 h, and the skin fully recovered within 4–8 days without evidence of necrosis or inflammation. These findings confirm that anti-BoNT/A antibody-integrated solid-to-gel microfilm-coated needles are well tolerated and biocompatible in vivo.

The safety of the anti-BoNT/A antibody-integrated solid-to-gel microfilm was evaluated in more detail (Figures S1–S4). Safety profiles were compared among groups treated with solid-state anti-BoNT/A, liquid anti-BoNT/A antibodies or saline. Physiological parameters including body weight, food intake, muscle strength, and motor coordination as well as relative organ weights, hematologic markers, and serum biochemical makers show similar patterns among the three groups, showing that the solid-state anti-BoNT/A antibody has no issues on safety profiles.

### 2.5. Pharmacokinetics and Protective Efficacy

**Figure 6:**
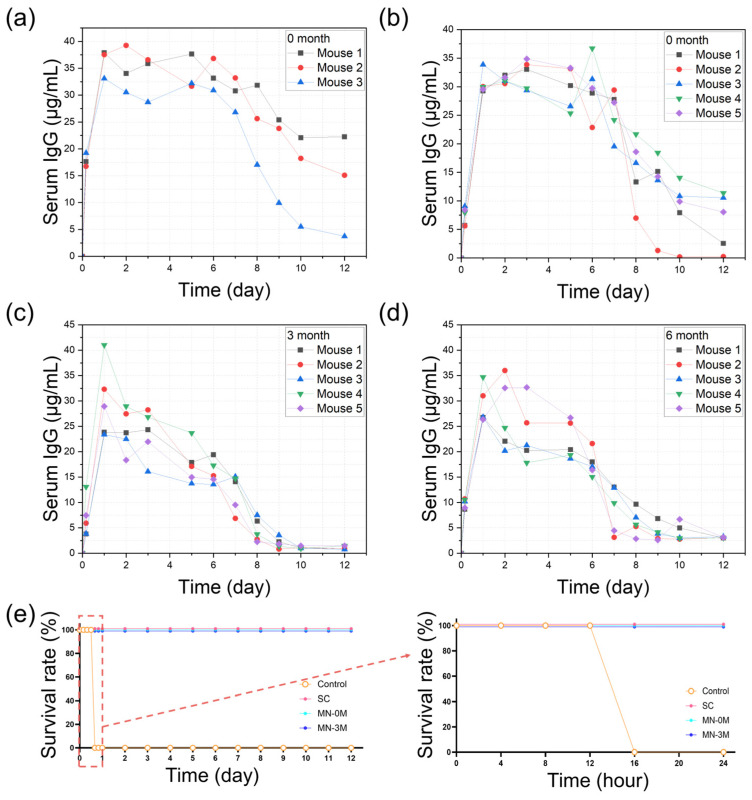
Pharmacokinetics and protective efficacy of anti-BoNT/A antibody-integrated solid-to-gel microfilm-coated needles. (**a**) Peripheral blood antibody concentrations following conventional subcutaneous injections of liquid antibodies (*n* = 3). (**b**–**d**) Peripheral blood antibody concentrations after delivery of solidified antibody stored for zero, three, or six months in ambient temperature, respectively (*n* = 5). (**e**) Survival analysis in a mouse BoNT/A challenge test, demonstrating protective efficacy of conventional subcutaneous injections of liquid antibodies (SC) and solid-state antibodies (stored for zero or three months in ambient temperature) compared with untreated controls. Survival of mice was observed at 4 h intervals up to 24 h, and then at 1-day intervals up to 12 days. The right panel shows detailed survival rates within 24 h. (Red box and arrow: 24-h observation results graph area).

Systemic bioavailability was evaluated by measuring serum antibody concentrations following conventional subcutaneous injections or anti-BoNT/A antibody-integrated solid-to-gel microfilm-coated needle application. Conventional subcutaneous liquid antibody injections produced rapid antibody appearance in peripheral blood, peaking within 24 h and gradually declining thereafter (Figure 6a). Freshly prepared antibody microfilm-coated needles (zero-month, Figure 6b) generated comparable blood antibody levels, confirming efficient skin absorption. Importantly, microfilm-coated needles stored under ambient conditions for three months (Figure 6c) and six months (Figure 6d) exhibited similar pharmacokinetic profiles, indicating preserved stability for efficient delivery to peripheral blood during long-term storage. Protective efficacy was validated in a lethal BoNT/A challenge model (Figure 6e). All untreated control mice (*n* = 5) succumbed within 16 h. In contrast, mice receiving conventional subcutaneous injections of liquid antibodies (*n* = 5, freshly dissolved in PBS) or solid-state antibodies stored for zero or three months (*n* = 5 per group) showed complete survival (100%) over the observation period up to 12 days following lethal BoNT/A challenge. These data establish that solid-state antibody application provides effective systemic protection equivalent to liquid antibody injections while retaining protective efficacy after prolonged storage.

The present study demonstrates that anti-BoNT/A antibody-integrated solid-to-gel microfilm-coated needles combine rapid subcutaneous delivery, long-term stability, and robust protective efficacy in vivo. Unlike conventional liquid formulations, which suffer from dose inaccuracy and pain during application and require cold-chain storage and logistics, the solidified antibody microfilm-coated needle format preserves antibody integrity for at least three to six months at room temperature, ensuring readiness for field deployment. The dissolution kinetics observed both in vitro and in vivo confirm that therapeutic antibody doses are delivered within 5 min, a critical timeframe for intervention against fast-acting bioterrorism agent such as BoNT/A. The comparison of pharmacokinetics between conventional liquid subcutaneous injections and microfilm-coated needle delivery underscores the reliability of this platform. Moreover, the challenge test results highlight its potential as an effective countermeasure in emergency or bioterrorism scenarios, where portability, stability, and ease of administration are paramount. Importantly, the absence of local tissue damage or systemic toxicity further supports the safety of this approach.

Taken together, these findings establish anti-BoNT/A antibody-integrated solid-to-gel microfilm-coated needles as a transformative strategy for antibody therapeutics, bridging the gap between laboratory efficacy and real-world deployment. Beyond BoNT/A, this platform may be readily adapted to other antibody-based therapeutics, enabling broad applications in infectious disease prevention, toxin neutralization, and biodefense. Future work will focus on scaling up production, extending stability validation beyond six months, and evaluating performance in larger animal models and ultimately in human clinical studies.

## 3. Conclusions

We developed a jetting-based fabrication platform for the uniform production of solid-state antibodies. By sequentially applying and low-temperature drying hyaluronic acid (HA) and anti-BoNT/A antibodies onto the metal needles, we generated structurally stable, concave-shaped antibody deposits that offer intrinsic advantages for subcutaneous anchoring. The anti-BoNT/A antibody-integrated solid-to-gel microfilm exhibited rapid dissolution within five minutes, as demonstrated by in vitro dissolution assays, and retained structural integrity after processing. In vivo evaluation in mouse models confirmed long-term stability over three to six months and therapeutic efficacy, as reflected by complete protection in challenge tests up to 12 days.

This solid antibody strategy enables accurate dosing, simplified storage and logistics, and rapid and safe administration, thereby expanding accessibility to emergency and field settings. Notably, its ease of use holds promise for self-administration by patients experiencing acute intoxication or those exposed to poisoning risks in wartime environments. By addressing the critical limitations of liquid antibody formulations, this approach has the potential to secure the therapeutic golden hour and improve survival outcomes. Although demonstrated here with BoNT/A antibodies, the platform may be broadly extended to diverse antibody therapeutics, paving the way toward a versatile solid-state antibody technology for biodefense and clinical medicine.

## 4. Materials and Methods

### 4.1. Materials

The anti-BoNT/A antibody solution was purchased Abion Bio Inc (Seoul, Republic of Korea). Hyaluronic acid (HA) powder (HA-TLM 20–40, 200–400 kDa) was purchased Bloomage Biotechnology Corporation (Jinan, China). The 23G metal needles (inner diameter 340 μm, outer diameter 640 μm) were from Korea Vaccine Co, Ltd., Seoul, Republic of Korea.

### 4.2. Fabrication Process

Anti-BoNT/A antibody solution was loaded into the syringe reservoir of the automated jetting system. Target-achieving area on the metal needles were designated using the integrated vision-guided positioning module. Prior to antibody deposition, the needle channels were pre-filled with a HA solution (0.2 mL, 1 *w*/*v*%), which gelled upon drying to serve as a biocompatible support matrix [27,28,29,30]. The pre-applied HA solution, after low-temperature drying, formed a solid coating that rapidly rehydrated upon contact with aqueous media, undergoing a swelling-driven gel-like transition that served as a biocompatible support for stable antibody anchoring and subsequent release. Antibody (0.1 mL, 3 *w*/*v*%) droplets of a defined volume (11.67 μL) were then dispensed sequentially onto the designated regions of the needles. A total of three sequential jetting cycles of BoNT/A antibody solution were conducted to fabricate a 100 μg solid-state antibody microfilm-coated needles. Each application was followed by low-temperature drying at 4 °C.

### 4.3. Surface and Compositional Analysis

The three-dimensional topography of solid-state anti-BoNT/A antibodies was characterized using a confocal microscope (Keyence, VR-6000, Itasca, IL, USA). Surface morphology was further examined by field-emission scanning electron microscopy (FE-SEM; FEI Sirion, Hillsboro, OR, USA). FE-SEM was conducted at an accelerating voltage of 5 kV with a working distance of 4 mm, using a secondary electron detector. Samples were either left uncoated or sputter-coated with a Pt layer of 5 nm depending on imaging requirements. FTIR spectroscopy (Transmission mode) was conducted over the spectral range of 500–4000 cm^−1^ with a resolution of 4 cm^−1^, averaging 32 scans per spectrum to assess the regional distribution of hyaluronic acid (HA) and antibody components within the solidified complexes.

### 4.4. Dissolution Assays

To evaluate dissolution kinetics, the anti-BoNT/A antibody-integrated solid-to-gel microfilm-coated needles were immersed in phosphate-buffered saline (PBS) at 37 °C for defined time intervals (30, 60, 120, 180, and 300 s). At each time point, the degree of antibody dissolution and the residual volume were quantified. Ultraviolet–visible spectroscopy (UV–vis; Perkin Elmer, Springfield, IL, USA) was employed for the quantitative analysis of antibody release. UV–vis spectroscopy with a 1 cm cuvette pathlength was employed to monitor antibody dissolution at 187 nm (and additionally at 280 nm), with measurements taken at 30, 60, 120, 180, and 300 s.

### 4.5. Ethical Approval and Animal Experiments

All animal procedures were approved by the Animal Care and Use Committee of the Agency for Defense Development (Approval No. ADD-IACUC-23-06). Female Balb/c mice, aged 6 weeks (Raon Bio, Seoul, Republic of Korea), were housed in groups of two or three per cage under standard conditions with free access to food and water. After a 1-week acclimatization period, antibody-coated needles were applied to the shaved dorsal skin of mice anesthetized with 40 mg/kg alfaxalone (Jurox, Rutherford, New South Wales, Australia) and 4 mg/kg xylazine (Bayer AG, Leverkusen, Germany). Each needle contained 100 μg of solidified anti-BoNT/A antibody coated on the tips. The needles were injected into the skin with gentle thumb pressure at a ~30° angle and maintained for 5 min to ensure complete dissolution of the solidified antibody coating. To assess skin response, photographs were taken immediately after injection and on days 1, 4, and 8 post-injection.

### 4.6. Pharmacokinetics Study

For pharmacokinetic analysis, mice were randomized into groups receiving either a subcutaneous injection of liquid anti-BoNT/A antibodies, microfilm-coated needle delivery of freshly prepared solid-state antibodies (0-month storage), the needle delivery after 3-month storage at 25 °C and 60% RH, or the needle delivery after 6-month storage at 25 °C and 60% RH. Blood samples (50–100 μL) were collected from the retro-orbital sinus at 4 h and 1—12 days post-injection. Samples were allowed to clot and centrifuged at 3000 rpm for 10 min to obtain a serum. Antibody concentrations were quantified using an ELISA specific for anti-BoNT/A IgG. Briefly, 96-well plates were coated overnight with purified BoNT/A antigen, blocked with 5% BSA in PBS, and incubated with serum samples diluted in PBS-Tween 20. Bound antibodies were detected with HRP-conjugated anti-human IgG secondary antibodies (Jackson ImmunoResearch, West Grove, PA, USA) and developed with TMB substrates. Absorbance was measured at 450 nm using a microplate reader (Molecular Devices, Sunnyvale, CA, USA), and antibody concentrations were calculated against a standard curve of purified anti-BoNT/A antibody.

### 4.7. BoNT/A Challenge Test

Mice were divided into groups (*n* = 5 per group): untreated control, SC antibody injection, MN antibody (0-month), and MN antibody (3-month). One day after treatment, mice were challenged by intraperitoneal injection of a lethal dose of BoNT/A (10× LD50). Mice were monitored every 4 h for 24 h and then every day for 12 days for signs of intoxication (ptosis, paralysis, respiratory distress) and survival.

## Data Availability

Data that supports the findings of this study are available from the corresponding authors upon request.

## Supplementary Materials

The following supporting information can be downloaded at: https://www.mdpi.com/article/10.3390/gels11100777/s1, Figure S1: Physiological parameters were monitored at two weeks after conventional subcutaneous injection (SC) of the liquid anti-BoNT/A antibody or microfilm-coated needle (MN) application of the solidified anti-BoNT/A antibody. The control group received a subcutaneous injection of saline. Body weight and food intake of each mouse were measured. Muscle strength was assessed using a grip strength meter. Motor coordination was evaluated using a rotarod tester (N = 6 for control group and MN group. N = 3 for SC group). Figure S2: Relative organ weights were compared among experimental groups at two weeks after conventional subcutaneous injection (SC) of the liquid anti-BoNT/A antibody or microfilm-coated needle (MN) application of the solidified anti-BoNT/A antibody. The control group received a subcutaneous injection of saline (N = 6 for control group and MN group. N = 3 for SC group). Figure S3: Hematological indices were compared among experimental groups at two weeks after conventional subcutaneous injection (SC) of the liquid anti-BoNT/A antibody or microfilm-coated needle (MN) application of the solidified anti-BoNT/A antibody. The control group received a subcutaneous injection of saline (N = 6 for control group and MN group. N = 3 for SC group). Figure S4: Serum biochemical makers were compared among experimental groups at two weeks after conventional subcutaneous injection (SC) of the liquid anti-BoNT/A antibody or microfilm-coated needle (MN) application of the solidified anti-BoNT/A antibody. The control group received a subcutaneous injection of saline (N = 6 for control group and MN group. N = 3 for SC group).
