# Supplementary material for: Antibody-Integrated Solid-to-Gel Microfilm for Protection Against Botulinum Neurotoxin Type A
Source: Gels. 2025 Sep 27;11(10):777. doi: 10.3390/gels11100777 (PMC12563112; doi:10.3390/gels11100777)
Supplement: Supplementary file 1 [file gels-11-00777-s001.zip › gels-3884294-supplementary.pdf]

Supplementary Data

To compare the safety profile of anti-BoNT/A antibody integrated solid-to-gel microfilm with conventional subcutaneous injection, several physiological parameters were monitored at 2 weeks after application. Body weight and food intake of each mouse were measured. Muscle strength was assessed using a grip strength meter (Bio-GS3, Bioseb, Vitrolles, France). Each mouse underwent six trials, with 1-minute rests between trials. The highest and lowest scores were excluded, and the remaining values were averaged. Motor coordination was evaluated using a rotarod tester (JD-A-07TSM, Jeung Do Bio, Seoul, Korea), where mice were placed on a rotating rod accelerating from 3 to 30 rpm over 5 minutes, and the retention time was recorded. After these assessments, mice were autopsied and organs were weighed. Relative organ weights were calculated by dividing organ weight by body weight of each mouse. Blood samples were collected through heart puncture and analyzed for complete blood count, including WBC, RBC, HGB, HCT, MCV, MCH, MCHC, PLT, RDW and MPV using a hematology analyzer (XN-1000V, Sysmex, Kobe, Japan). Then, serum samples were obtained by centrifugation and analyzed for serum biochemistry, including albumin (ALB), alkaline phosphatase (ALP), aspartate transaminase (AST), blood urea nitrogen (BUN), creatinine, total protein, glucose, total cholesterol, and triglycerides using a serum biochemistry analyzer (AU480, Beckman Coulter, Brea, CA, USA).

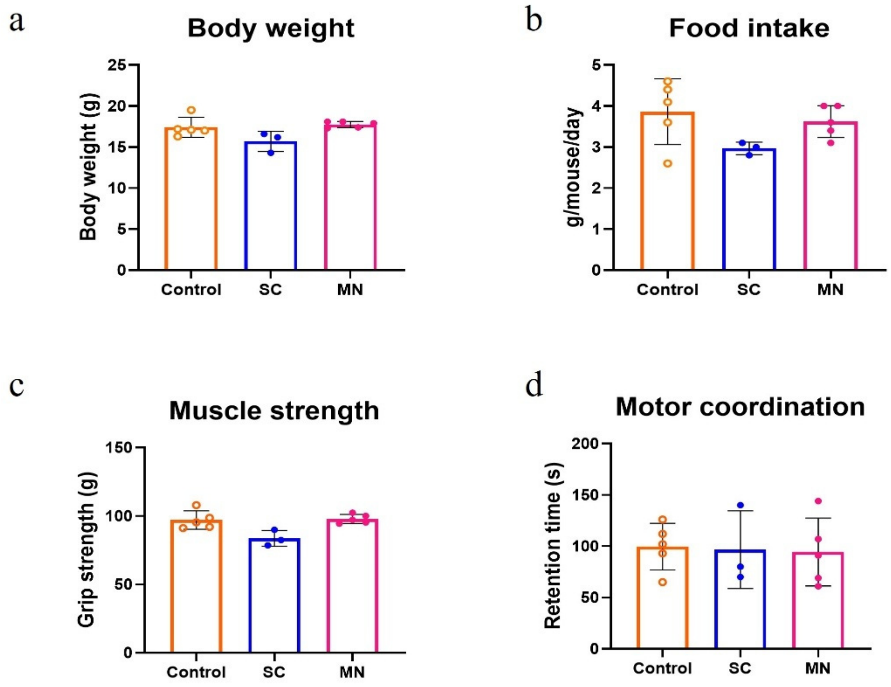

Figure S1. Physiological parameters were monitored at 2 weeks after conventional subcutaneous injection (SC) of liquid anti-BoNT/A antibody or microfilm-coated needle (MN) application of solidified anti-BoNT/A antibody. Control group received subcutaneous injection of saline. Body weight and food intake of each mouse were measured. Muscle strength was assessed using a grip strength meter. Motor coordination was evaluated using a rotarod tester (N=6 for control group and MN group. N=3 for SC group).

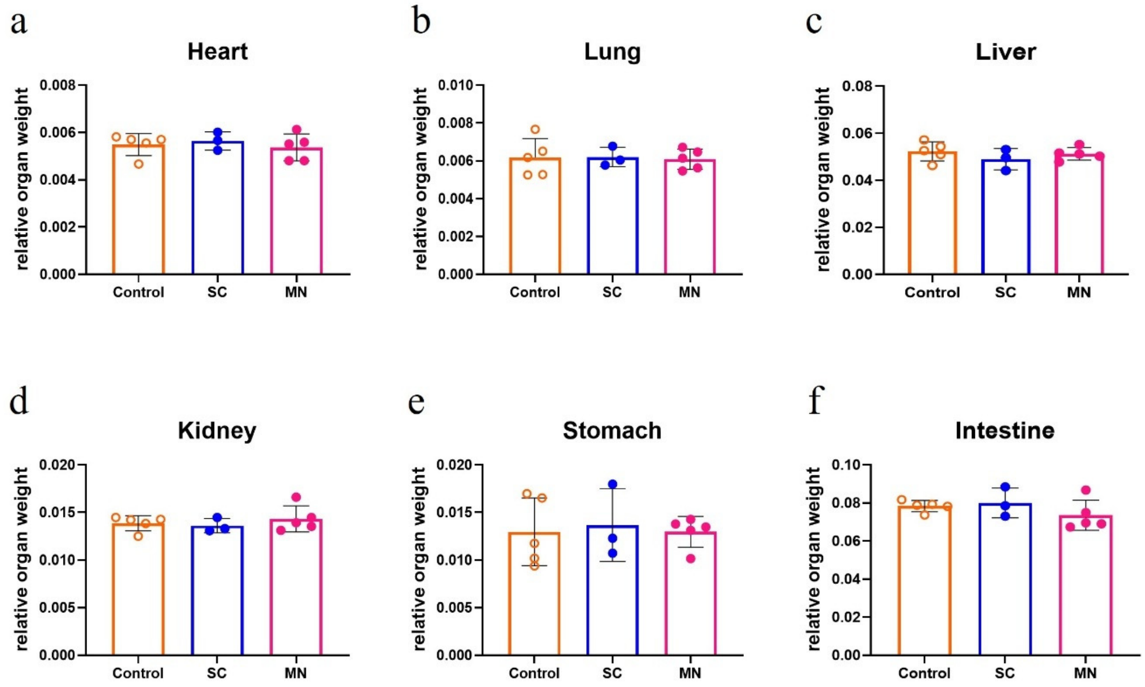

Figure S2. Relative organ weights were compared among experimental groups at 2 weeks after conventional subcutaneous injection (SC) of liquid anti-BoNT/A antibody or microfilm-coated needle (MN) application of solidified anti-BoNT/A antibody. Control group received subcutaneous injection of saline (N=6 for control group and MN group. N=3 for SC group).

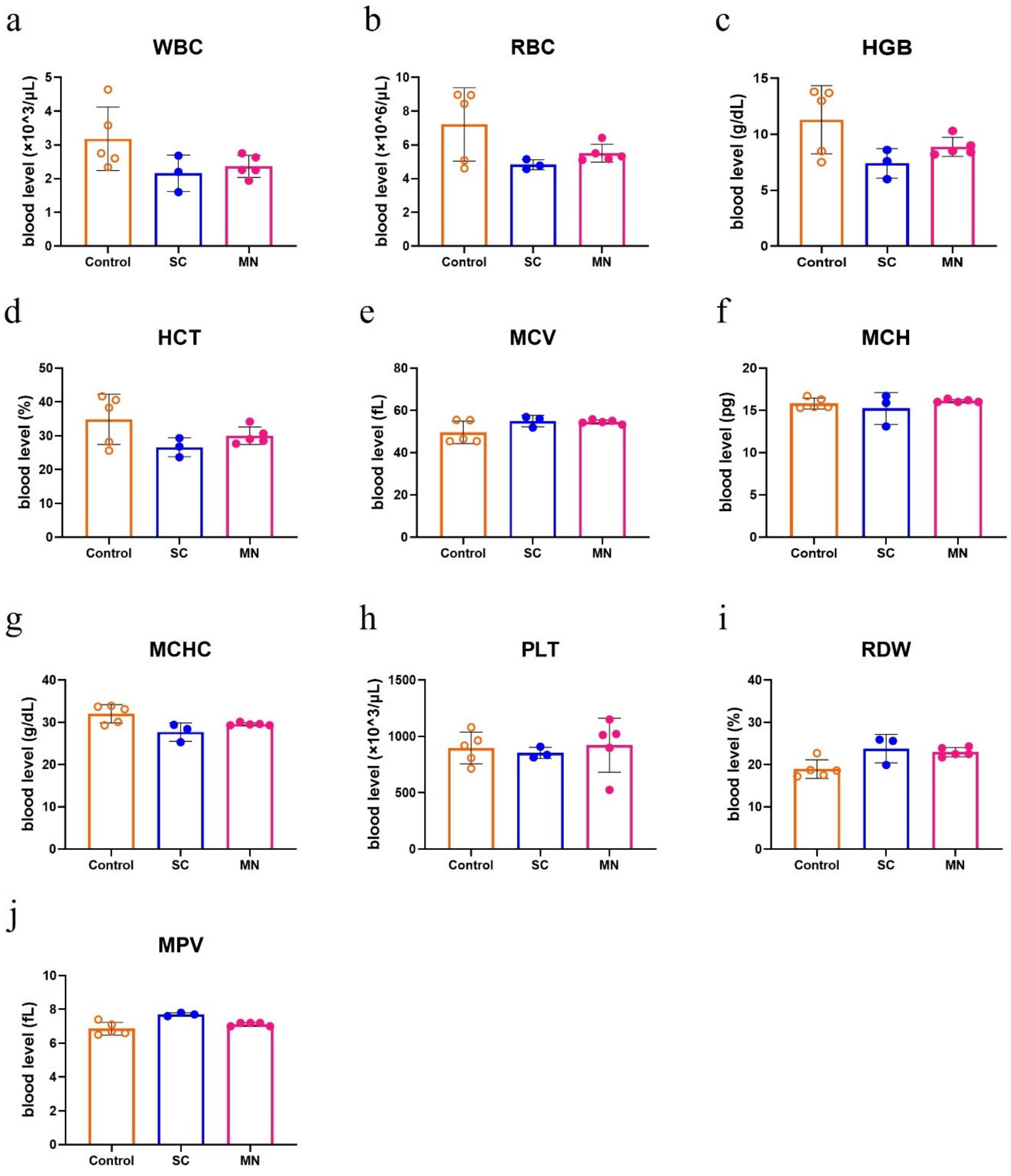

Figure S3. Hematological indices were compared among experimental groups at 2 weeks after conventional subcutaneous injection (SC) of liquid anti-BoNT/A antibody or microfilm-coated needle (MN) application of solidified anti-BoNT/A antibody. Control group received subcutaneous injection of saline (N=6 for control group and MN group. N=3 for SC group).

WBC: white blood cell, RBC: red blood cells, HGB: hemoglobin, HCT: hematocrit, MCV: mean corpuscular volume, MCH: mean corpuscular hemoglobin, MCHC: mean corpuscular hemoglobin concentration, PLT: platelet, RDW: red cell distribution width, MPV: mean platelet volume

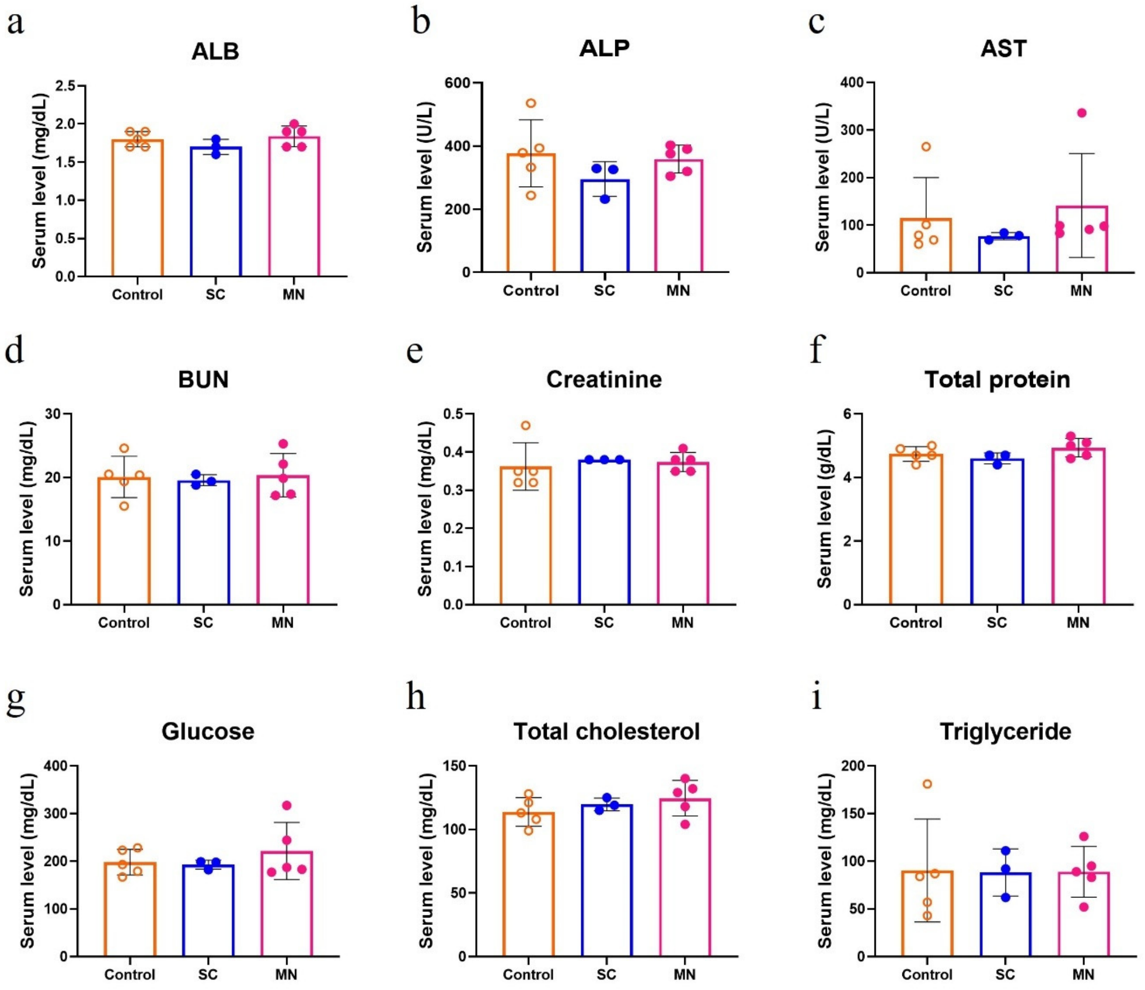

Figure S4. Serum biochemical makers were compared among experimental groups at 2 weeks after conventional subcutaneous injection (SC) of liquid anti-BoNT/A antibody or microfilm-coated needle (MN) application of solidified anti-BoNT/A antibody. Control group received subcutaneous injection of saline (N=6 for control group and MN group. N=3 for SC group).

ALB: albumin, ALP: alkaline phosphatase, AST: aspartate transaminase, BUN: blood urea nitrogen,
